# Inflammatory myofibroblastic tumor of the mesentery with a *SQSTM1::ALK* fusion responding to alectinib

**DOI:** 10.1002/cnr2.1792

**Published:** 2023-02-08

**Authors:** Cass G. G. Sunga, Michael S. Higgins, Robert W. Ricciotti, Yajuan J. Liu, Lee D. Cranmer

**Affiliations:** ^1^ Division of Medical Oncology, Department of Medicine University of Washington School of Medicine Seattle Washington USA; ^2^ PeaceHealth Department of General and Colorectal Surgery Bellingham Washington USA; ^3^ Department of Laboratory Medicine and Pathology University of Washington School of Medicine Seattle Washington USA; ^4^ Clinical Research Division Fred Hutchinson Cancer Center Seattle Washington USA

**Keywords:** alectinib, anaplastic lymphoma kinase, inflammatory myofibroblastic tumor, sequestosome 1

## Abstract

**Background:**

Inflammatory myofibroblastic tumor (IMT) is an ultra‐rare soft tissue neoplasm associated with fusion proteins encompassing the anaplastic lymphoma kinase (ALK) protein fused to a variety of partner proteins. Data regarding response to ALK‐targeting agents based on fusion partner is limited.

**Case:**

A 30‐year‐old female sought emergency care after onset of abdominal and lower back pain in 2019. Computed tomography (CT) demonstrated a cystic, mesenteric mass within the pelvis measuring up to 8.9 cm. Complete laparoscopic excision of the mass from the mesentery of the right colon and terminal ileum was performed. Pathologic assessment revealed IMT with a fusion between sequestosome 1 and ALK (*SQSTM1::ALK*), described in only two other cases of IMT. Four months after surgery, CT revealed multi‐focal, unresectable disease recurrence. She was referred to the University of Washington/Fred Hutchinson Cancer Center and placed on therapy with alectinib, after which she experienced a partial response. Three years after IMT recurrence, disease remains under control.

**Conclusion:**

This is the third reported case of IMT associated with the novel SQSTM1::ALK fusion protein, and the second treated with alectinib. Treatment with the ALK inhibitor alectinib appears to be active in this setting.

## 1 INTRODUCTION

Inflammatory myofibroblastic tumor (IMT) is a rare mesenchymal neoplasm predominantly found in children and young adults; in 75% of cases, the condition occurs in the abdominal cavity, such as the mesentery, omentum, and retroperitoneal space.^1^ A variety of other sites may be affected, including head/neck, lungs, bladder, central nervous system, and ovaries/uterus. While patients may present with symptoms associated with mass effects from the primary tumor, IMT may also have a variety of vague and non‐specific signs and symptoms, reflecting its “inflammatory” behavior, such as fevers, fatigue and weight loss.^1^, ^2^ Laboratory assessments may demonstrate an elevated erythrocyte sedimentation rate (ESR) or C‐reactive protein, thrombocytosis, anemia, leukocytosis, and hypergammaglobulinemia, phenomena potentially related to paraneoplastic expression of interleukin‐6.^2^, ^3^ First‐line treatment for IMT is complete surgical resection, if possible, with the possibility of re‐resection for local recurrence. No formal clinical trials of conventional cytotoxic therapies have been conducted in IMT; retrospective analyses demonstrate some responses, although no clear pattern of benefit.^4^

Despite recognition of the condition decades ago, IMT was conflated with a benign, reactive process now known as inflammatory pseudotumor, which has no malignant potential.^1^, ^5^ Observation of recurrent translocations at 2p23 with identification of the anaplastic lymphoma kinase (*ALK*) gene in fusion proteins led to the recognition that IMT cases with these alterations have intermediate malignant potential.^6^, ^7^, ^8^ Translocations involving *ALK* may be present in 50%–60% of IMT cases, although there is heterogeneity.^1^, ^4^, ^9^ In cases with absent *ALK* protein expression or translocation, additional target genes, such as *ROS1, NTRK, RET*, and *PDGFR‐beta*, have been identified.^9^

In its native state, ALK is a transmembrane receptor tyrosine kinase that peaks in expression during the first 3 weeks of life. Its baseline physiologic role is poorly understood, with some animal models suggesting a role in fetal nervous system development.^10^, ^11^ A specific ligand for the receptor has not been identified. ALK activation leads to activity in several downstream pathways, including Janus kinase‐signal transducer and activator of transcription, RAS‐mitogen activated protein kinase, and the phosphoinositide‐3‐kinase‐Akt‐mammalian target of rapamycin pathways.

*ALK* was initially discovered as an oncogene in anaplastic large‐cell lymphoma and has been subsequently implicated in a number of malignancies, including non‐small cell lung cancer (NSCLC), large B‐cell lymphoma, and renal cell carcinoma. ALK fusion proteins contain the intracellular tyrosine kinase signaling domain, with the N‐terminal component of the fusion developing from any of a variety of fusion partners. The fusion presumably conveys constitutive activation of the kinase.

Identification of *ALK* translocations in IMT prompted clinical trials with crizotinib, a first‐generation ALK inhibitor.^12^, ^13^ These were necessarily small in size, but demonstrated clear activity of the drug in those with *ALK* translocation, versus markedly lesser benefit in those without *ALK* translocations. Crizotinib, while an effective therapy, is limited by poor penetration through the blood–brain barrier, toxicity, and development of resistance.^14^ This prompted the development of second‐generation ALK inhibitors, including alectinib, designed to overcome these challenges.

To highlight the utility of second‐generation ALK inhibitors in targeting a unique fusion protein, we here present a case of a 30‐year‐old female diagnosed with IMT who subsequently developed multi‐focal disease recurrence. Tumoral cells possessed a gene fusion of the N‐terminal portion of sequestosome 1 (*SQSTM1*) and the C‐terminal of *ALK*, designated *SQSTM1::ALK*, which has only been reported in two other cases of IMT.^9^, ^15^ She demonstrated marked disease regression and prolonged disease control after treatment with alectinib.

## 2 CASE

**FIGURE 1:**
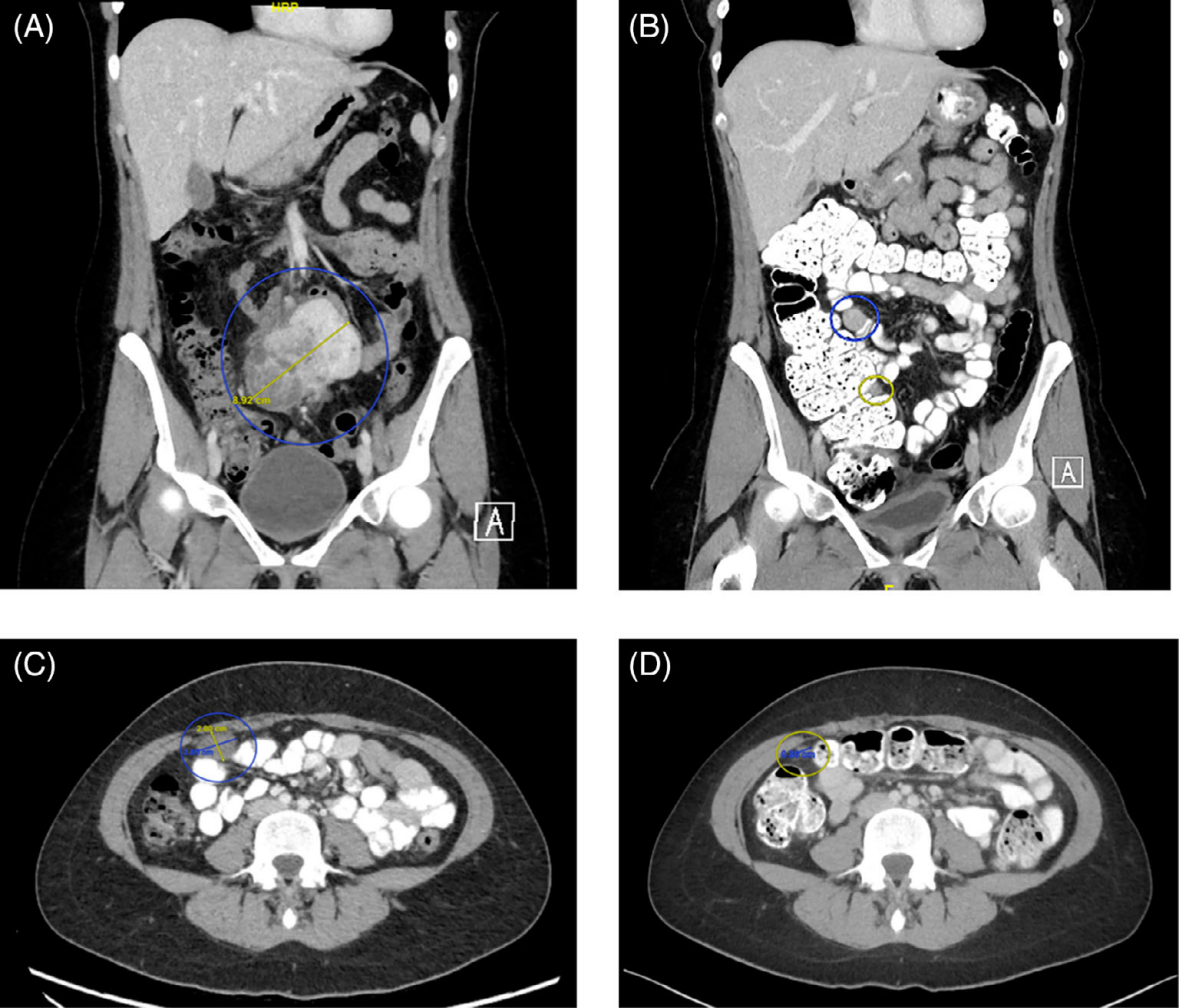
Computed tomography of the abdomen and pelvis. (A) Coronal images at time of diagnosis, demonstrating an 8.9 cm mesenteric mass. (B) Coronal images obtained 4 months after resection, with circles indicating sites of disease recurrence. (C) Axial image obtained 3 months after initiation of alectinib treatment. A 2 cm tumor mass is circled. (D) Axial image at similar level as that in image (C), obtained 14 months after initiation of alectinib treatment. The previously noted mass is now less than 1 cm maximal dimension.

**FIGURE 2:**
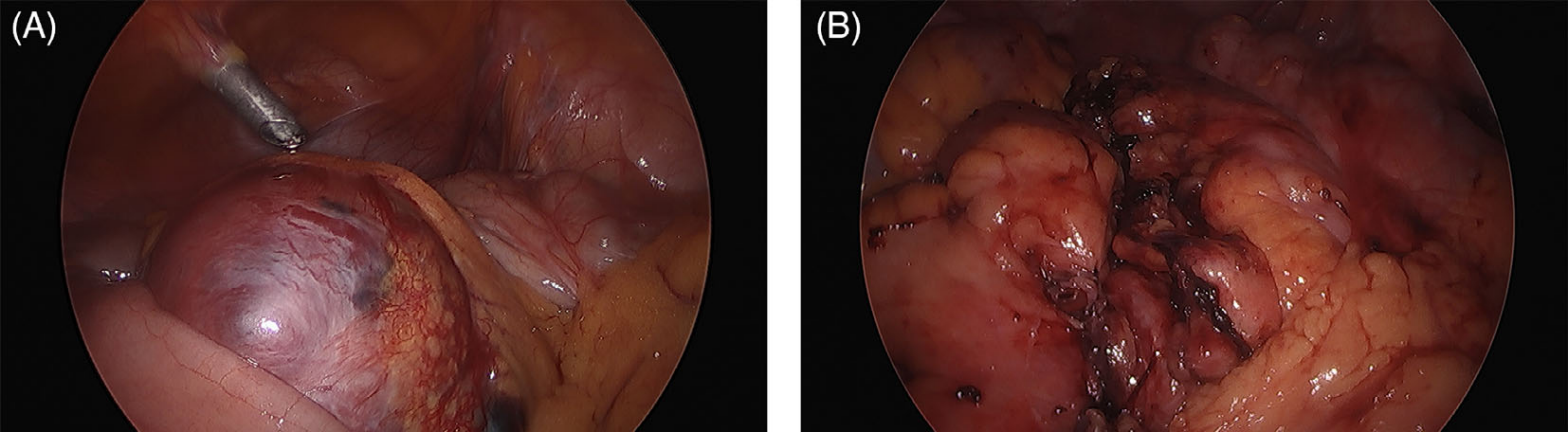
Images obtained during complete laparoscopic resection. (A) Mesenteric mass in situ. (B) Mesentery after tumor and small bowel resection.

A 30‐year‐old female with no significant medical comorbidities or history sought emergent care for progressive abdominal and lower back pain in 2019. She was evaluated by her primary care doctor prior to this encounter and was found to have an ESR of 81 mm/hr (normal <23 mm/hr). Computed tomography (CT) of her abdomen and pelvis demonstrated a cystic, mesenteric pelvic mass measuring up to 8.9 cm in maximal dimension (Figure 1A). Complete laparoscopic excision of the mass was performed from the mesentery of the right colon and terminal ileum (Figure 2).

**FIGURE 3:**
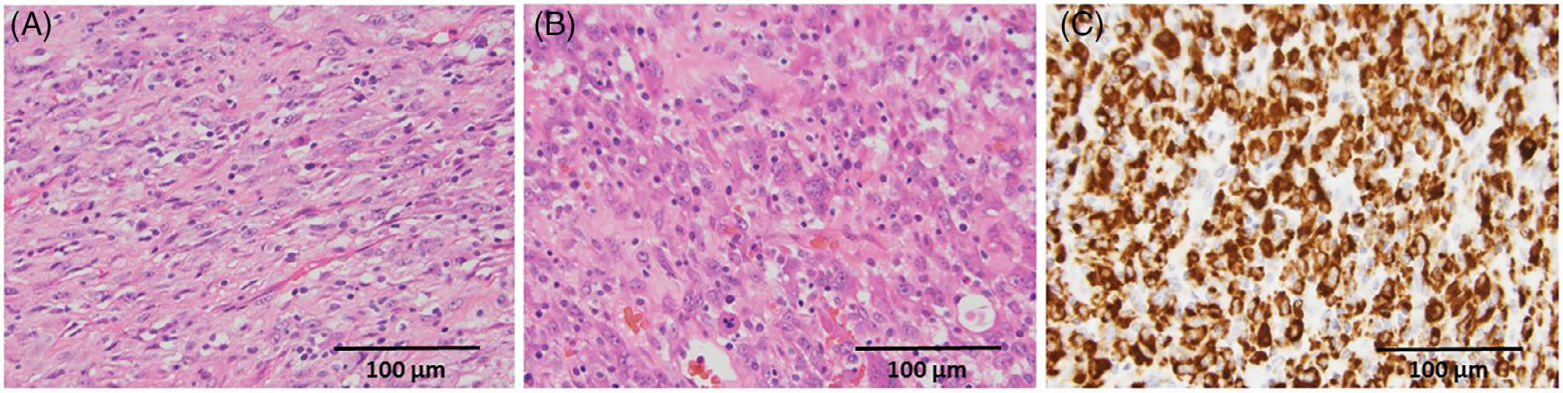
Histopathology of mesenteric mass obtained after resection. (A) Histologic appearance of the neoplasm demonstrating eosinophilic spindle cells with background mixed inflammatory infiltrate typical of inflammatory myofibroblastic tumor (H&E, 400×). (B) An area of the neoplasm demonstrating more epithelioid cells with greater nuclear pleomorphism within a somewhat myxocollagenous matrix. Mitotic figures, including an atypical tripolar mitosis, are present (H&E, 400×). (C) Immunohistochemical staining for ALK expression using a D5F3 antibody clone shows diffuse cytoplasmic expression (400×).

Histologic sections showed a neoplastic proliferation of spindled‐to‐epithelioid cells with variably pleomorphic nuclei, some of which were very large with irregular contours and prominent nucleoli (Figure 3A). The cells were within a background of variably dense collagenous‐to‐myxoid stroma with an intermixed inflammatory infiltrate (Figure 3B). There was scattered mitotic activity, including occasional atypical mitoses, but no tumoral necrosis. Immunohistochemical (IHC) stains demonstrated diffusely positive cytoplasmic staining for ALK in the neoplastic cells using a D5F3 antibody clone, but only focal weak staining using an ALK1 antibody clone (Figure 3C). FISH testing was negative for *MDM2* gene amplification and in situ hybridization studies were negative for kappa and lambda light chain expression and Epstein–Barr virus. Additional IHC was negative for markers of hematolymphoid differentiation in the spindled and epithelioid cells. *SQSTM1::ALK* fusion was detected by anchored multiplex polymerase chain reaction and RNA‐seq with custom 115‐gene FusionPlex solid tumor panel.

**FIGURE 4:**
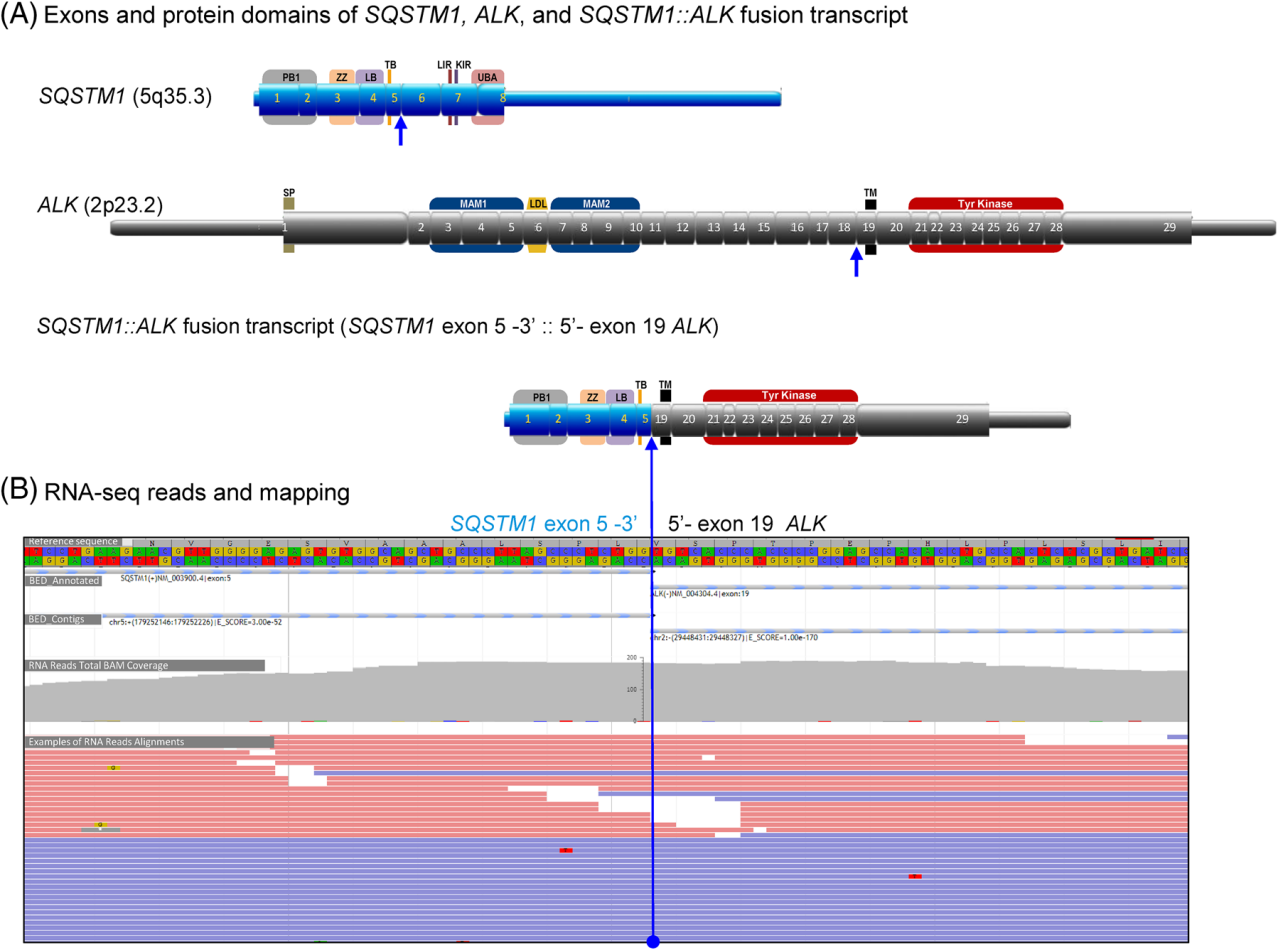
*SQSTM1::ALK* fusion identified by RNA‐seq. (A) The structures of *SQSTM1* (blue boxes), *ALK* (gray boxes), and *SQSTM1::ALK* fusion transcript with their functional protein domains. *SQSTM1::ALK* fusion transcript has 3′ end of exon 5 of *SQSTM1* fused (::) with 5′ end of exon 19 of *ALK*. Untranslated regions (5′ UTR and 3′ UTR) are shown as narrow bars. Exons are shown as boxes with numbers. The keys of protein domains are shown in the wider boxes behind exons with letters: for the domain structure of SQSTM1/p62 protein, KIR, Keap1‐interacting region; LB, LIM protein‐binding; LDL, low‐density lipoprotein receptor domain class A; LIR, LC3‐interacting region; MAM, meprin, A5 protein, and Mu domain; PB1, Phox and Bem1p; SP, signal peptide; TB, TRAF6‐binding domain; TM, transmembrane region; Tyr Kinase, tyrosine kinase domain; UBA, ubiquitin‐associated; for the domain structure of ALK protein; ZZ, Zinc finger. The blue lines and arrows indicate the breakpoints and fusion points. (B) Representative sequence reads over the breakpoints of the *SQSTM1::ALK* fusions by paired‐end RNA sequencing, including exon mapping of chimeric transcripts to the reference sequence at base resolution with amino acid translation in frame. Total BAM coverages of the breakpoints (~200 reads) were shown as gray bar scale. Examples of next‐generation sequencing reads over the breakpoints were shown in red bars (R1 reads) and blue bars (R2 reads).

The predicted fusion involves a translocation of chromosome 2 involving the 3′ end of the *ALK* gene (NM_004304) to chromosome 5 involving the 5′ end of the *SQSTM1* gene (NM_003900) (Figure 4). The *SQSTM1::ALK* fusion transcript is in‐frame between 3′ end of exon 5 of *SQSTM1* (NM_003900) and 5′ end of exon 19 of *ALK* (NM_004304). The resulting fusion protein is composed of exons 1–5 of SQSTM1 (amino acids 1 to 251) in the N‐terminal and exons 19–29 of ALK (amino acids 1023 to 1620) in the C‐terminal. This 849 amino acid fusion polypeptide retained the four main functional domains of SQSTM1 (PB1, ZZ, LB, and TB domains) and the transmembrane domain and tyrosine kinase domain of ALK (Figure 4). The 5′ fusion partner, SQSTM1, is ubiquitously expressed with total median expression of 1628.86 RPKM (Figure 4A). Under SQSTM1 promoter, the SQSTM1::ALK fusion gene most likely leads to constitutive expression of the protein, with consequent activation of the ALK kinase domain.

She was referred to the University of Washington/Fred Hutchinson Cancer Center for monitoring and management. Four months after resection, the patient reported no symptoms. However, surveillance CT showed local recurrence in the mesentery and omentum, and metastatic disease in the extraperitoneal space anterior to the bladder (Figure 1B). Given the multifocal nature of the recurrence, surgical resection was judged to be infeasible and systemic therapy was recommended. Due to the identification of a unique ALK translocation in this patient, targeted therapy with alectinib was initiated at a dose of 600 mg twice daily. CT scans were obtained every 3 months thereafter to monitor for disease progression (Figure 1C).

Highlighted below, the patient's disease burden stabilized while on alectinib and showed substantial improvement at 14 months (Figure 1D). Imaging 3 years into treatment shows essentially stable disease with no new metastases. Throughout this entire time, the patient was able to tolerate treatment with no significant adverse events, other than fatigue. She continued to maintain her employment undertaking administrative work during treatment.

## 3 DISCUSSION

We present a case of a 30‐year‐old female patient with mesenteric IMT complicated by metastatic recurrence. In the primary resection specimen, IHC demonstrated diffuse positive staining for ALK using a D5F3 antibody clone. The presence of an ALK fusion protein, specifically SQSTM1::ALK, was documented, consistent with the IMT family of tumors. Other diagnoses considered on presentation included retroperitoneal dedifferentiated liposarcoma and a hematolymphoid neoplasm, which were excluded with negative testing for *MDM2* amplification and additional IHC studies for hematolymphoid markers, respectively.

Several observations suggested that this patient might be affected by a variant of epithelioid inflammatory myofibroblastic sarcoma (EIMS).^16^, ^17^, ^18^ These findings included its mesenteric site of origin, rapid multi‐focal recurrence after initial surgical treatment, and the focal epithelioid cytology with severe cytologic atypia and occasional atypical mitotic figures. EIMS is reported to have more aggressive clinical behavior, and has been associated, although not exclusively, with the RANBP2‐ALK fusion protein.^16^ A nuclear or peri‐nuclear ALK IHC staining pattern, reported in association with the RNABP2 partner, was not present in our patient; its lack does not exclude the clinical diagnosis of EIMS.

Instead, an ALK fusion protein with an N‐terminal derived from SQSTM1 was identified. This has been reported in two other cases of IMT.^9^, ^15^ In one patient, the primary tumor originated in the right subclavian region and progressed slowly over the course of 5 years until margin‐negative resection. Multi‐focal recurrent disease was identified 12 months later. This patient, similar to ours, received alectinib treatment with partial response and controlled disease after 12 months. This behavior was similar to that seen in our patient and suggests that the *SQSTM1::ALK* fusion is associated with a more aggressive clinical course. A second patient was reported to have this fusion and was treated for 4.2 months with crizotinib, achieving stable disease.^9^

At present, crizotinib is the only drug approved by the US Food and Drug Administration for treatment of ALK‐positive IMT.^19^ Use of other ALK inhibitors for IMT is consistent with treatment recommendations from the National Comprehensive Cancer Network.^20^ Alectinib has been compared directly to crizotinib in NSCLC.^21^, ^22^ In NSCLC, alectinib is associated with improved progression‐free survival and better safety/tolerability than crizotinib. Alectinib has additional theoretical advantages, including activity against ALK mutants selected during crizotinib therapy.^14^ Due to its better tolerability and theoretical advantages over crizotinib, we have used alectinib in this and other IMT patients in our clinic. Crizotinib could however have been used as therapy in this condition, as reported in one other case possessing this fusion, albeit treated for only 4.2 months.^9^

SQSTM1::ALK fusions have been identified in several other clinical conditions associated with ALK fusion proteins. These include non‐neural granular cell tumors, ALK‐positive B‐cell lymphoma, epithelioid fibrous histiocytoma of the skin, and NSCLC.^23^, ^24^, ^25^, ^26^, ^27^, ^28^, ^29^ Neither the clinical nor histologic characteristics of our patient would suggest one of these alternate diagnoses.

*SQSTM1* encodes a highly conserved 62 kDa protein (also known as p62) which exerts effects as a main regulator of multiple signaling pathways, including Nrf2, mTORC1, and NF‐κB.^30^ It has been associated with a variety of malignancies, and non‐malignant conditions, including Paget's disease of bone, amyotrophic lateral sclerosis, and steatohepatitis and alcoholic hepatitis. Studies of the SQSTM1::ALK fusion protein in B‐cell lymphoma have suggested that the STAT3 pathway may be a viable therapeutic target.^24^ As early as 2002, it was suggested that therapeutic strategies targeting ALK and its downstream effectors, including STAT3, might represent effective approaches in ALK‐driven malignancies.^31^ A recent pre‐clinical report identified synergistic activity against ALK‐fusion‐dependent H2228 cells when the orally bioavailable STAT3 inhibitor YHO‐1701 was used in combination with alectinib.^32^ STAT3 also has a complex relationship with endogenous SQSTM1. The addition of quercetin, a STAT3‐inhibiting flavonoid, leads to decreased accumulation and activity of SQSTM1 and decreased Epstein Barr virus‐mediated B cell immortalization.^33^ It would be worth exploring whether such a combination ALK/STAT3‐inhibitor strategy may be applicable in IMT.

In conclusion, we report a patient with IMT associated with an SQSTM1::ALK fusion protein, which has been reported in only two other cases. As in one of the prior patients, the disease has responded to treatment with alectinib, and continues to do so 3 years after diagnosis of recurrent disease. This provides evidence of the utility of this therapy in this clinical setting, and potentially in other malignancies in which this rare, but recurrent fusion protein target is found.

## AUTHOR CONTRIBUTIONS

**Cass G. G. Sunga:** Conceptualization (equal); methodology (equal); visualization (equal); writing – original draft (equal); writing – review and editing (equal). **Michael S. Higgins:** Resources (equal); writing – review and editing (equal). **Robert W Ricciotti:** Methodology (equal); resources (equal); visualization (equal); writing – original draft (equal); writing – review and editing (equal). **Yajuan J Liu:** Methodology (equal); resources (equal); visualization (equal); writing – review and editing (equal). **Lee Cranmer:** Conceptualization (equal); methodology (equal); resources (equal); supervision (lead); writing – original draft (equal); writing – review and editing (equal).

## CONFLICT OF INTEREST STATEMENT

The authors have stated explicitly that there are no conflicts of interest in connection with this article.

## ETHICS STATEMENT

The reporting of this case does not meet the definition of “research” according to 45 CFR 46.102d. Approval by Human Subjects Protection is not required. Written informed consent was obtained directly from the patient for the publication of case details and use of images. All activities were consistent with the Declaration of Helsinki. Work submitted has been performed according to Wiley's Publication Ethics Guidelines with no data fabrication or misconduct.

## Data Availability

Data sharing is not applicable to this article as no new data were created or analyzed in this study.
